# Head-to-head comparison of ^18^F-FDG and ^18^F-FES PET/CT for initial staging of ER-positive breast cancer patients

**DOI:** 10.1186/s41824-023-00176-3

**Published:** 2023-12-18

**Authors:** Peerapon Kiatkittikul, Supanida Mayurasakorn, Chetsadaporn Promteangtrong, Anchisa Kunawudhi, Dheeratama Siripongsatian, Natdanai Hirata, Attapon Jantarato, Natphimol Boonkawin, Sukanya Yaset, Pattanapong Kongsakorn, Warunya Phewnual, Chanisa Chotipanich

**Affiliations:** National Cyclotron and PET Centre, Chulabhorn Hospital, Chulabhorn Royal Academy, 906 Kamphaeng Phet 6 Rd., Lak Si, Bangkok, 10210 Thailand

**Keywords:** ^18^F-FES, ^18^F-FDG, ^18^F-fluroestradiol, ^18^F-flurodeoxyglucose, ER-positive breast cancer, Initial staging

## Abstract

**Purpose:**

To compare the diagnostic performance of ^18^F-fluorodeoxyglucose (^18^F-FDG) and ^18^F-fluoroestradiol (^18^F-FES) positron emission tomography/computed tomography (PET/CT) for initial staging of estrogen receptor (ER) positive breast cancer.

**Methods:**

Twenty-eight patients with ER-positive breast cancer underwent ^18^F-FDG and ^18^F-FES PET/CT for initial staging. Diagnostic performance and concordance rates were analyzed for both radiotracers. Semiquantitative parameters of maximum standardized uptake value (SUVmax) and tumor-to-normal ratio (T/N ratio) were compared using Wilcoxon signed-rank test. Factors potentially affecting the degree of radiotracer uptake were analyzed by multi-level linear regression analysis.

**Results:**

The overall diagnostic performance of ^18^F-FES was comparable to ^18^F-FDG, except for higher specificity and NPV, with sensitivity, specificity, PPV, NPV, and accuracy of 87.56%, 100%, 100%, 35.14%, and 88.35%, respectively, for ^18^F-FES and 83.94%, 30.77%, 94.74%, 11.43%, and 95.37%, respectively, for ^18^F-FDG. Diagnostic performance of strong ER expression was better in ^18^F-FES but worse for ^18^F-FDG. There was a correlation of mucinous cell type and Allred score 7–8 with ^18^F-FES uptake, with correlation coefficients of 26.65 (19.28, 34.02), 5.90 (− 0.005, 11.81), and *p*-value of < 0.001, 0.05, respectively. Meanwhile, luminal B and Ki-67 were related to ^18^F-FDG uptake, with correlation coefficients of 2.76 (1.10, 0.20), 0.11 (0.01, 0.2), and *p*-value of 0.018, 0.025, respectively.

**Conclusion:**

Diagnostic performance of ^18^F-FES is comparable to ^18^F-FDG, but better for strongly ER-positive breast cancer. Combination of ^18^F-FES and ^18^F-FDG would potentially overcome the limitations of each tracer with more accurate staging.

## Introduction

Breast cancer is the most common cancer in woman, accounting for 24.2% of all cancers in women, and the most frequent cause of cancer-related death in women, at 15% (Ferlay et al. 2019). The prognosis and management of breast cancer depends on TNM staging and estrogen receptor (ER) expression. Approximately 75% of women with breast cancer have ER-positive tumors (Blamey et al. 2010). The evaluation of ER relies on immunohistochemistry (IHC) testing, which requires tissue biopsy, a more invasive procedure that may not be available in some regions.

Positron emission tomography/computed tomography (PET/CT) is an evaluation tool that is especially useful in cancer patients, providing functional information at the molecular level. ^18^F-fluorodeoxyglucose (^18^F-FDG) is a glucose analog that reflect the metabolic activity of the tumor cell. ^18^F-FDG PET/CT is widely used in the evaluation of various cancers, including breast cancer. However, it has several limitations, such as infection or inflammation, resulting in false-positive lesions (Boellaard et al. 2015).

^18^F-fluoroestradiol (^18^F-FES) is an estrogen analog and an FDA-approved radiotracer for PET scans (Research C for DE and Drug Trial Snapshot: CERIANNA 2020). ^18^F-FES can selectively bind to ER in cancer cells, especially breast cancer, and exhibits a good correlation to the degree of ER expression detected by IHC (Gupta et al. 2017; Mintun et al. 1988). Thus, ^18^F-FES PET can be used for non-invasive evaluation of the ER in the whole body. This study aimed to compare the diagnostic performance of ^18^F-FDG and ^18^F-FES PET/CT in the initial staging of ER-positive breast cancer.

## Materials and methods

### Study design

This was a retrospective, single-center comparative imaging study, approved by the Human Research Ethics Committee of Chulabhorn Research Institute, with no external source of funding. The primary objective was to compare the diagnostic performance of ^18^F-FDG and ^18^F-FES PET/CT in the initial staging of ER-positive breast cancer. The secondary objective was to identify the concordance rate between ^18^F-FDG and ^18^F-FES PET/CT, including potential factors affecting the degree of ^18^F-FDG and ^18^F-FES uptake.

This study recruited all breast cancer patients who underwent PET/CT scan at National Cyclotron and PET Centre, Chulabhorn Hospital, Bangkok, Thailand, from 1 September 2020 to 31 October 2022. The inclusion criteria were patients aged > 18 years with pathologically confirmed ER-positive breast cancer. The exclusion criteria were those with fasting blood sugar > 200 mg/dL, a history of other cancers, known ER negativity, unknown ER status, and pregnancy or breastfeeding.

The demographic data collection included age, sex, body mass index (BMI), menopausal status, and tissue pathology results. ER expression was defined according to: (i) luminal A (Ki-67 < 14%) and luminal B (Ki-67 > 14%) subtype (Network 2023); and (ii) Allred score calculated from the summation of intensity and proportion scores of ER expression (range, 0–8). The Allred score was further classified as negative (score 0–2), intermediate (score 3–6), or high (score 7–8) (Weischenfeldt et al. 2017).

### Imaging protocol

^18^F-FDG and ^18^F-FES PET/CT were performed on different days within 2-week interval. The patients were advised to avoid any meals for at least 4–6 h and strenuous exercise for 24 h prior to ^18^F-FDG PET/CT, while there was no specific preparation for ^18^F-FES PET/CT. The plasma glucose level was tested before ^18^F-FDG PET/CT. If higher than 200 mg/dL, ^18^F-FDG PET/CT was postponed. The intravenous injection dose of ^18^F-FDG was calculated according to patient’s body weight (2.59 MBq/kg), but a fixed dose was used in ^18^F-FES (111 MBq). After 60-min radiotracer administration, PET scan was acquired from vertex to proximal thigh using a 64-slice Siemens/Biograph Vision PET/CT scanner (Siemens Healthcare GmbH, Erlangen, Germany) in the three-dimensional mode with continuous bed motion method at speed of 1.6–1.8 mm/s. The matrix was 440 × 440, with the reconstruction methods of True X and Time of Flight. The CT parameters were 120 kV tube voltage, 25 mAs current, and 3 mm slice thickness.

### Image analysis

^18^F-FDG and ^18^F-FES PET/CT scans were separately interpreted by three board-certified nuclear medicine physicians with consensus. ^18^F-FDG PET/CT were reviewed by P.K., A.K., and C.P. The ^18^F-FES PET/CT were reviewed by P.K., D.S., and C.C. The images were reviewed using Syngo.via workstation (Siemens Healthcare GmbH). The physicians were blinded to clinical data at the time of review.

Image analysis was based on visual detection. An area of focal uptake higher than the surrounding background indicated a positive lesion. The lesions were assessed as primary tumor (T stage), regional nodal metastases (N stage), and distant metastasis (M stage) based on the eighth edition of the American Joint Committee on Cancer Staging System for breast cancer (Amin et al. 2017). A maximum of seven lesions were acquired in each region. Regional nodal metastasis was classified into axillary level I, level II, level III, and supraclavicular node. Non-regional node metastasis, brain, visceral organ in the chest and abdomen, bone, and soft tissue involvement were individual sites for distant metastasis.

Three-dimensional voxels of interest (VOI) were drawn around the lesions, with semiquantitative parameters acquired by three designated physicians. The VOIs were manually adjusted by the physicians to avoid false-positive regions caused by normal physiological uptake. The maximum standardized uptake value (SUVmax) was determined in all lesions. The tumor-to-normal ratio (T/N ratio) of all lesions were calculated by dividing SUVmax of the lesion with SUVmean of the mediastinal blood pool.

### Reference standard

Tissue histopathology with immunohistochemistry staining is the gold standard for diagnostic accuracy analysis. For non-biopsied lesions, the reference standard was anatomically observed on CT or magnetic resonance imaging (MRI). For nodal metastasis, there was a cluster of at least three size-independent nodes at one site or fewer than three lymph nodes with at least one measuring ≥ 1 cm along the short axis or spherical form or central necrosis. For lung metastasis, a solid pulmonary nodule, reticulonodular pattern, cavitating nodule, or lymphangitis carcinomatosis was included. For bone metastasis, an osteolytic or sclerotic lesion with cortical breakthrough, periosteal reaction, expansile appearance, or pathological fracture observed by CT or an abnormal marrow signal on MRI were considered. For other distant metastasis, a nodule or mass lesion not compatible with benign lesion was considered.

### Statistical analysis

Demographic data from all patients are presented as number, percentage, mean ± SD (standard deviation), or median with interquartile range (IQR). The concordance rates were calculated between both radiotracers. Diagnostic accuracy was defined by sensitivity, specificity, positive predictive value (PPV), negative predictive value (NPV), and accuracy. Differences in semiquantitative parameters were analyzed using Wilcoxon signed-rank test. Potential factors affecting the degree of uptake for both radiotracers were identified by multi-level linear regression analysis. A *p*-value of < 0.05 was considered statistically significant. STATA software, version 11 (Stata Corp LLC; College Station, TX, USA) was applied for statistical analyses.

## Results

Sixty-four patients underwent PET/CT scan of both radiotracers for initial staging in breast cancer. Of these, 13 patients were excluded due to ER negativity, followed by 15 with an indication of complete staging after surgery and 8 of unknown ER status. Thus, 28 female patients were included, most of whom were in menopause (78.57%) and who had a mean age of 59.1 ± 13.23 years, and mostly normal BMI (67.86%) at 22.10 ± 3.29 kg/m^2^. In pathological results, 20 patients (71.43%) had invasive ductal carcinoma (IDC), followed by 5 (17.86%) invasive lobular carcinoma (ILC), 1 (3.57%) invasive micropapillary carcinoma, and 1 (3.57%) mucinous carcinoma. The Allred scoring system showed 18 patients with score of 8, followed by 1 (score of 7), 2 (score of 5), 3 (score of 3), and 4 unknown score due to lack of data. For IHC results, 5 (17.86%) were luminal A, followed by 18 (64.28%) luminal B, and 5 unknown due to lack of data (Table 1).

**Table 1:** Baseline characteristics

Baseline data (28)	Results
Age (mean ± SD)	59.1 ± 13.23
*Menopause (n, percent)*	
Yes	22 (78.57%)
No	4 (21.43%)
BMI (mean ± SD)	22.10 ± 3.29
Underweight (< 18.5)	1 (3.57%)
Normal (18.5–24.9)	19 (67.86%)
Overweight (25.0–30)	7 (25%)
Obesity (> 30)	1 (3.57%)
*Cell type (n, percent)*	
Invasive ductal carcinoma (IDC)	20 (71.43%)
Invasive lobular carcinoma (ILC)	5 (17.86%)
Invasive micropapillary carcinoma	1 (3.57%)
Mucinous carcinoma	1 (3.57%)
*Allred Score (n, percent)*	
3	3 (10.71%)
5	2 (7.14%)
7	1 (3.57%)
8	18 (64.29%)
N/A	4 (14.29%)
*Luminal (n, percent)*	
A	5 (17.86%)
B	18 (64.28%)
N/A	5 (17.86%)

*BMI* Body mass index, *N/A* Not applicable, *SD* Standard deviation

**Fig. 1:**
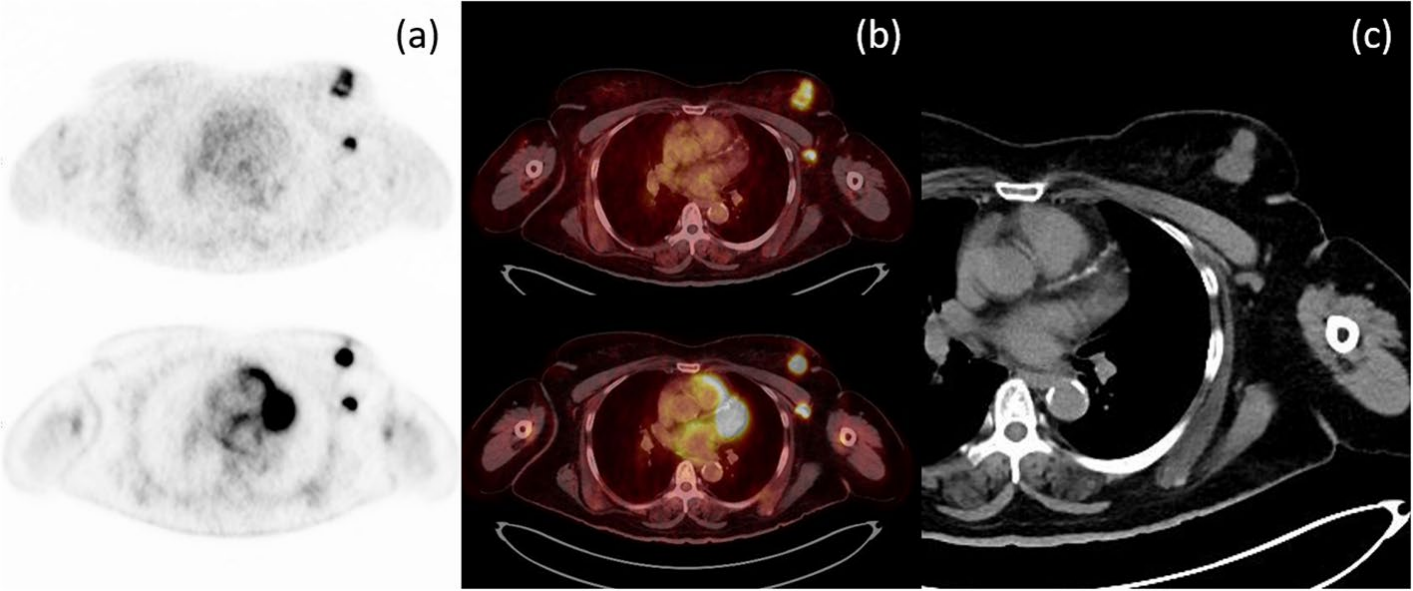
Axial PET (**a**) and axial fusion (**b**) images of ^18^F-FES (above) and ^18^F-FDG (below) with concordant uptake of both radiotracers in a left breast mass and left axillary node metastasis, in correlation with CT imaging (**c**)

**Fig. 2:**
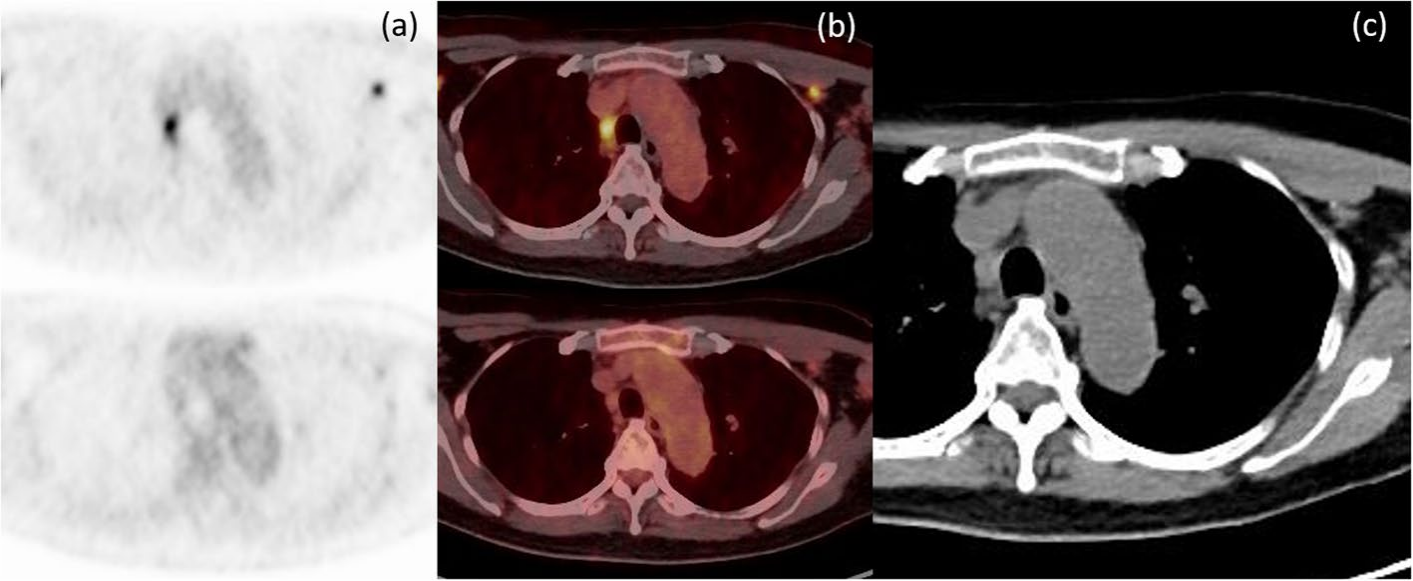
Axial PET **a** and axial fusion **b** images of ^18^F-FES (above) and ^18^F-FDG (below) for ^18^F-FES-avid enlarged nodes on CT imaging **c** without ^18^F-FDG avidity at left axillary and right paratracheal nodes

**Fig. 3:**
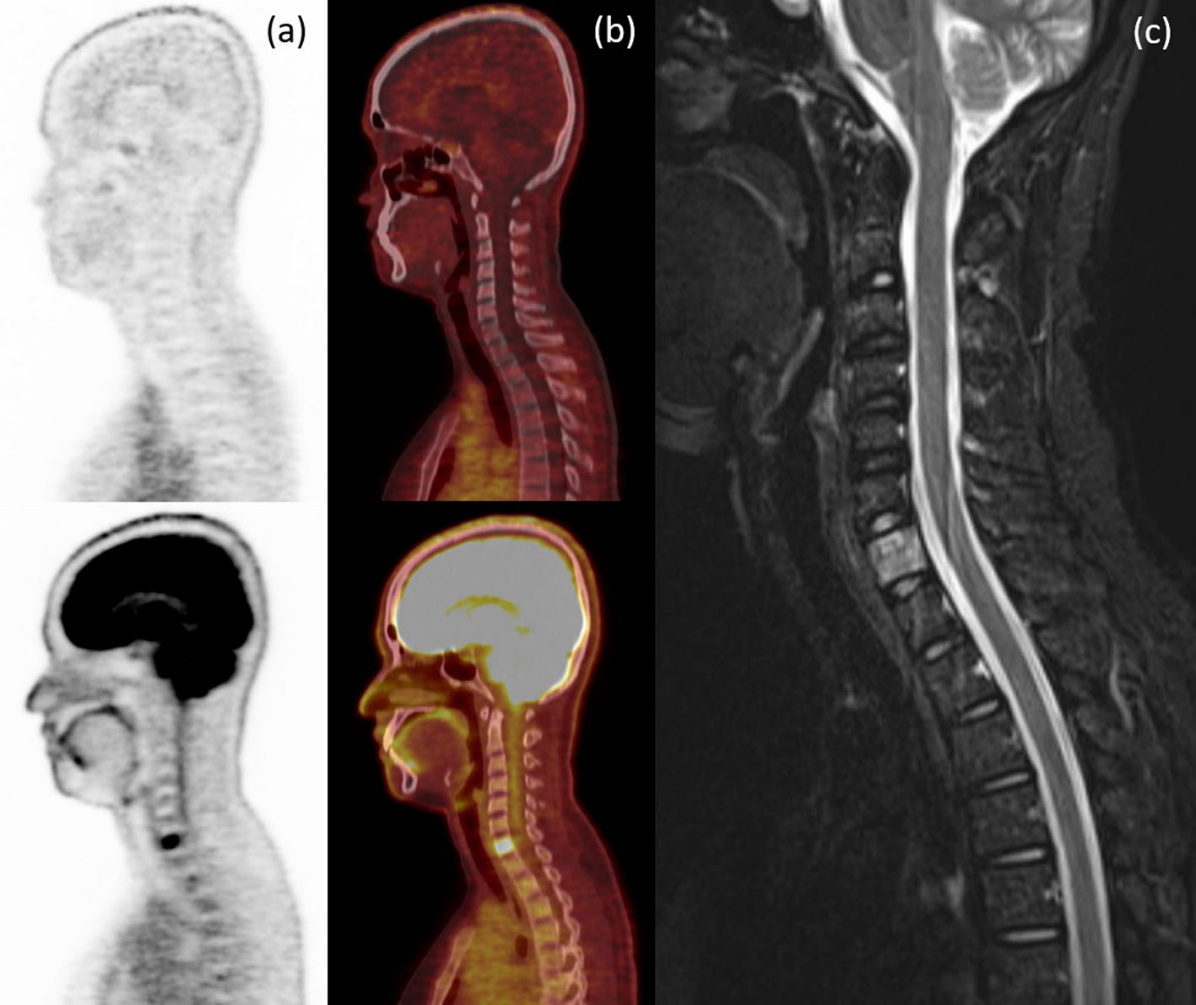
Sagittal PET **a** and sagittal fusion **b** images of ^18^F-FES (above) and ^18^F-FDG (below) for ^18^F-FDG-avid hyperT2 bone metastasis at C7 vertebra on MRI (**c**) without ^18^F-FES avidity

**Fig. 4:**
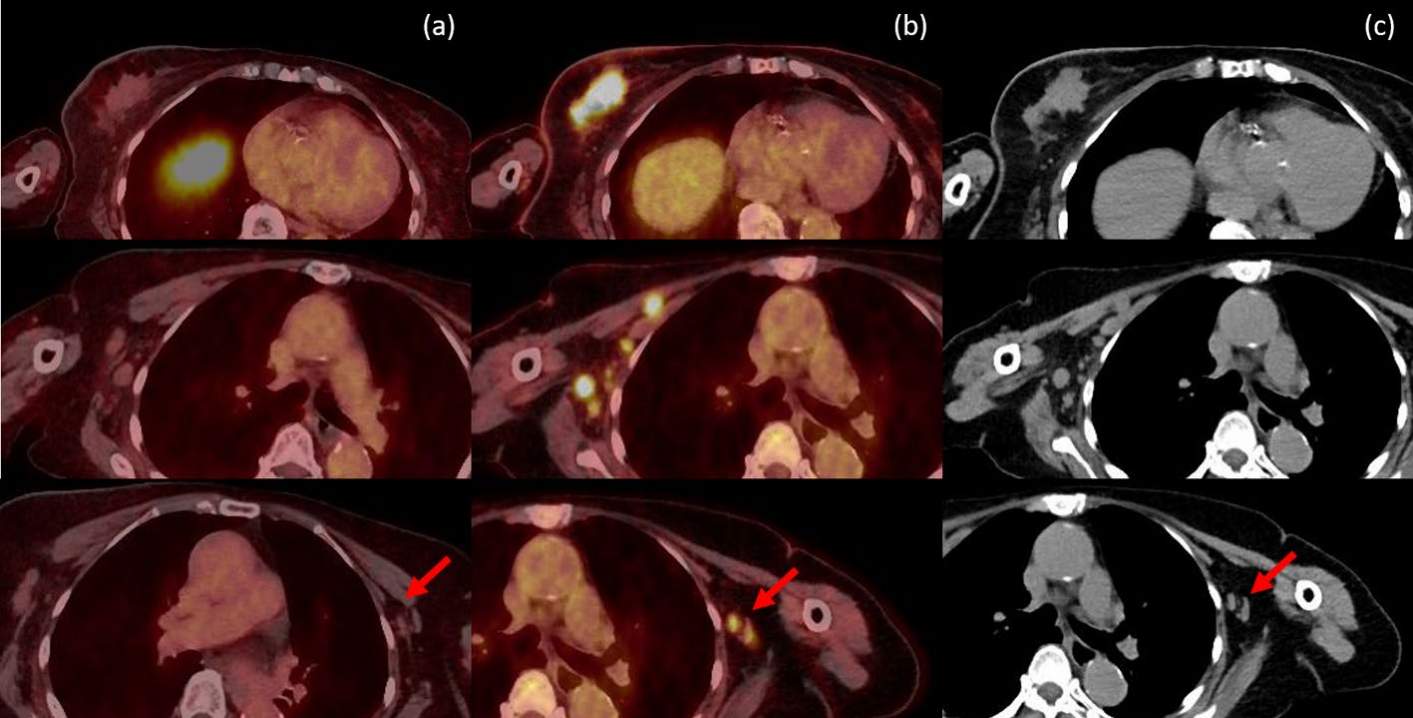
Fusion PET axial images of ^18^F-FES **a** and ^18^F-FDG **b** for ^18^F-FDG-avid right breast mass and multiple enlarged metastatic nodes at right axilla on CT imaging **c** without ^18^F-FES avidity, in line with few small ^18^F-FDG-avid nodes at left axilla (arrow) but no ^18^F-FES avidity compatible with reactive nodes from recent vaccination and decreased size in follow-up imaging

For patient analysis (Table 2), two patients (7.14%) had discordant results between ^18^F-FDG and ^18^F-FES PET/CT in T stage. One had falsely downstage from ^18^F-FDG PET/CT and the other had falsely downstage from ^18^F-FES PET/CT. In N stage, 7 patients (25%) had discordant results, including 1 falsely upstage from ^18^F-FDG, 4 falsely downstage from ^18^F-FDG, and 2 falsely downstage from ^18^F-FES. In M stage, 6 patients (21.43%) had discordant results, namely 3 falsely upstage in ^18^F-FDG (all from non-regional reactive nodes), 2 falsely downstage in ^18^F-FDG (one non-regional lymph node and the other with bone metastases), and 1 falsely downstage in ^18^F-FES (bone metastasis). In overall TNM stage, 18 patients (64.29%) had concordant results (Fig. 1). Among 10 discordant results, there were 3 false positive of ^18^F-FDG PET/CT with increased TNM stage, 5 false negative of ^18^F-FDG PET/CT with decreased TNM stage (Fig. 2), 1 false negative of ^18^F-FES PET/CT with decreased TNM stage (Fig. 3), and 1 both false positive ^18^F-FDG and false negative ^18^F-FES PET/CT (Fig. 4).

**Table 2:** TNM staging of ^18^F-fluorodeoxyglucose and ^18^F-fluoroestradiol PET/CT

Cases	^18^F-flurodeoxyglucose PET/CT				^18^F-fluoroestradiol PET/CT			
	T	N	M	Stage	T	N	M	Stage
1	1	0	0	1	1	0	0	1
2	1	1**	0	2a	1	0	0	1
3	2	1	0	2b	2	1	0	2b
4	4	0*	0*	3b	4	1	1	4
5	2	0	0	2a	2	0	0	2a
6	4	0	1**	4	4	0	0	3b
7	4	1	0	3b	4	1	0	3b
8	4	0	0	3b	4	0	0	3b
9	4	3	1	4	4	3	1	4
10	2	3	1	4	2	3	1	4
11	2	3	1	4	2	3	1	4
12	4	3	1**	4	4	3	0	3c
13	4	3	1	4	4	3	1	4
14	2	1	0*	2b	2	1	1	4
15	3	3	0	3c	3	3	0	3c
16	1	0*	0	1	1	1	0	2a
17	2	3	1**	4	0*	0*	0	0
18	3	2	1	4	3	2	1	4
19	0*	0*	0	0	1	1	0	2a
20	2	0	0	2a	2	0	0	2a
21	2	1	0	2b	2	1	0	2b
22	1	1	0	2a	1	1	0	2a
23	2	0	0	2a	2	0	0	2a
24	2	1	1	4	2	0*	0*	2a
25	2	0*	0	2a	2	1	0	2b
26	2	1	1	4	2	1	1	4
27	2	0	0	2a	2	0	0	2a
28	2	0	0	2a	2	0	0	2a

*False negative lesion, resulting in down staging, ** False positive lesion, resulting in up staging

*PET/CT* Positron emission tomography/computed tomography

In the lesion analysis, there were a total of 206 lesions with 193 lesions of true metastasis. These included 54 T stage, 85 N stage, and 67 M stage. For M stage, there were 20 non-regional lymph nodes, 27 bone lesions, 19 lung lesions, and 1 adrenal gland lesion. ^18^F-FDG PET detected 171 lesions with true metastasis in 162 lesions. ^18^F-FES PET detected 169 lesions with true metastasis in all lesions.

The diagnostic performance of ^18^F-FES PET/CT achieved 87.56% sensitivity, 100% specificity, 100% PPV, 35.14% NPV, and 88.35% accuracy. Meanwhile, the diagnostic performance of ^18^F-FDG PET/CT exhibited 83.94% sensitivity, 30.77% specificity, 94.74% PPV, 11.43% NPV, and 80.58% accuracy. Further subgroup analysis in strong ER expression group (Allred score of 7–8), there was an increase in diagnostic performance of ^18^F-FES with 94.95% sensitivity, 100% specificity, 100% PPV, 64.29% NPV, and 95.37% accuracy. In contrast, there was a decrease in diagnostic performance of ^18^F-FDG with 72.73% sensitivity, 44.44% specificity, 93.51% PPV, 12.9% NPV, and 70.37% accuracy.

The semiquantitative parameters of all lesions showed statistical significance for high T/N ratio of ^18^F-FES when compared with ^18^F-FDG, with median (IQR) of 3.335 (1.61–6.38) and 2.635 (1.58–4.79), respectively (*p* = 0.016). However, there was no statistically significant difference in SUVmax of ^18^F-FES and ^18^F-FDG, with median (IQR) of 5.28 (2.55–10.60) and 5.68 (3.41–10.03), respectively (*p* = 0.646). For subgroup analysis, ILC cell type, Allred score 7–8, and luminal A category yielded a statistically significant increase degree of ^18^F-FES uptake in both SUVmax (*p* = 0.028, *p* = 0.003, and *p* = 0.019, respectively) and T/N ratios (*p* = 0.015, *p* < 0.001, and *p* = 0.004, respectively). In contrast, a significant increase in ^18^F-FDG uptake was noted for Allred score < 7 (*p* < 0.001 for both SUVmax and T/N ratio) and luminal B subtype (*p*-value = 0.008 for SUVmax) (Table 3).

**Table 3:** Difference in degree of uptake between ^18^F-fluorodeoxyglucose and ^18^F-fluoroestradiol

	Median (interquartile range)		*p*-value
	^18^F-flurodeoxyglucose	^18^F-fluoroestradiol	
All lesion			
SUVmax	5.68 (3.41–10.03)	5.38 (2.55–10.60)	0.646
T/N ratio	2.635 (1.58–4.79)	3.335 (1.61–6.38)	0.016
IDC cell type			
SUVmax	6.23 (3.63–10.34)	5.23 (2.29–10.6)	0.138
T/N ratio	3.14 (1.63–5.05)	3.26 (1.55–6.43)	0.144
ILC cell type			
SUVmax	2.15 (1.24–4.25)	4.75 (2.76–8.78)	0.028
T/N ratio	0.96 (0.55–1.79)	3.30 (1.92–6.09)	0.015
Allred score			
SUVmax	4.06 (2.20–6.42)	6.51 (3.51–9.69)	0.003
T/N ratio	1.92 (1.22–2.96)	3.57 (1.97–5.89)	< 0.001
Score < 7			
SUVmax	9.49 (5.22–17.66)	1.72 (1.34–2.79)	< 0.001
T/N ratio	4.96 (3.00–9.37)	1.48 (0.81–2.03)	< 0.001
Luminal Subtype			
Luminal A			
SUVmax	1.92 (1.33–11.13)	3.44 (2.76–6.77)	0.019
T/N ratio	0.86 (0.56–0.96)	2.18 (1.92–3.98)	0.004
Luminal B			
SUVmax	7.39 (3.79–11.13)	5.43 (2.34–10.60)	0.008
T/N ratio	3.56 (1.69–5.05)	3.24 (1.40–6.38)	0.718

*IDC* Invasive ductal carcinoma, *ILC* Invasive lobular carcinoma, *SUVmax* Maximal standardize uptake value, *T/N ratio* Tumor-to-normal ratio

Several factors significantly affected the degree of ^18^F-FDG PET in breast cancer. Luminal B subtype and Ki-67 were related to the degree of ^18^F-FDG uptake with correlation coefficient of 2.76 (95%CI 1.10,11.92), *p* = 0.018 and 0.11 (95%CI 0.01,0.20), *p* = 0.025, respectively. In contrast to ^18^F-FES, mucinous carcinoma cell type and Allred score of 7–8 were statistically significant with correlation coefficient of 26.65 (95%CI 19.28, 34.02), *p*-value < 0.001 and 5.90 (95%CI − 0.0005, 11.81), *p*-value = 0.05, respectively (Table 4).

**Table 4:** Factors associated with degree of uptake in ^18^F-fluorodeoxyglucose and ^18^F-fluoroestradiol PET/CT

Factor	^18^F-fluorodeoxyglucose		^18^F-fluoroestradiol	
	Correlation coefficient (95% C.I.)	*p*-value	Correlation coefficient (95% C.I.)	*p*-value
Age (year)	0.01 (− 0.14, 0.16)	0.937	0.12 (− 0.08, 0.31)	0.233
BMI (kg/m^2^)	0.16 (− 0.37, 0.69)	0.551	0.45 (− 0.21, 1.12)	0.181
*Menopause status*				
No	Ref		Ref	
Yes	− 0.33 (− 5.30, 4.65)	0.898	5.74 (− 0.54, 12.01)	0.073
Cell type				
Invasive ductal carcinoma	Ref		Ref	
Invasive lobular carcinoma	− 5.32 (− 11.48, 0.84)	0.090	1.46 (− 4.26, 7.19)	0.616
Invasive micropapillary carcinoma	− 0.95 (− 10.24, 8.33)	0.841	− 0.64 (− 8.24, 6.95)	0.868
Mucinous carcinoma	− 5.04 (− 14.18, 4.10)	0.280	26.65 (19.28, 34.02)	< 0.001
*Intensity of ER expression*				
Weak	Ref		Ref	
Moderate	− 1.87 (− 10.22, 6.48)	0.660	0.34 (− 10.21, 10.90)	0.949
Strong	− 3.82 (− 9.04, 1.40)	0.152	5.83 (− 0.56, 12.22)	0.074
*Proportion of ER expression*				
< 1%	–		–	
1-10%	Ref		Ref	
11-33%	0.22 (− 10.34, 10.79)	0.967	− 1.33 (− 14.63, 11.96)	0.844
34-66%	− 0.64 (− 8.03, 6.75)	0.865	3.20 (− 6.00, 12.41)	0.495
> 67%	− 2.39 (− 7.87, 3.09)	0.393	5.84 (− 0.75, 12.43)	0.082
*Allred Score*				
< 7	Ref		Ref	
7–8	− 3.60 (− 8.38, 1.18)	0.140	5.90 (− 0.0005, 11.81)	0.050
*Luminal*				
A	Ref		Ref	
B	2.76 (1.10, 11.92)	0.018	− 1.39 (− 5.11, 2.34)	0.465
Ki-67	0.11 (0.01, 0.20)	0.025	− 0.06 (− 0.12, 0.01)	0.096

*BMI* Body mass index, *ER* Estrogen receptor

## Discussion

Accurate initial TNM staging is a crucial step for appropriate management and prediction of prognosis in breast cancer (Network 2023). In this study, ^18^F-FES PET/CT could detect true metastatic lesions better than ^18^F-FDG PET/CT, as reported by previous studies (Liu et al. 2019; Ulaner et al. 2021; Piccardo et al. 2022; Chae et al. 2020). Our results yielded an overall diagnostic performance of ^18^F-FES that was comparable to previous studies (Gupta et al. 2017; Piccardo et al. 2022; Chae et al. 2019; Venema et al. 2017; Yang et al. 2013a; Kurland et al. 2020). ^18^F-FES had remarkably high selective binding to ER. There was no report of any false-positive finding from ^18^F-FES PET/CT, except one case report of a false-positive from post-radiation pneumonia (Yang et al. 2013b), resulting in a remarkably high PPV of 100% in our study. Thus, lesions with ^18^F-FES uptake were ER-positive metastasis. However, this study showed a low NPV of ^18^F-FES. This result could be explained by heterogeneity of ER-expression in the tumor with dissimilar expression of ER throughout the whole body (Turashvili and Brogi 2017; Babayan et al. 2013). ^18^F-FDG PET/CT detects glucose metabolism of tumor cells, with higher uptake in more aggressive tumors. In this study, the diagnostic performance of ^18^F-FDG PET/CT was also comparable to recent studies (Gupta et al. 2017; Liu et al. 2019; Piccardo et al. 2022; Chae et al. 2020), but lower in specificity and NPV when compared with ^18^F-FES PET/CT. These findings could be explained by the histological cell type of low-grade breast cancer such as ILC, with false negative in ^18^F-FDG PET/CT (Kumar et al. 2009) and non-specific uptake of ^18^F-FDG PET/CT, such as infection and inflammation (Boellaard et al. 2015).

In a subgroup analysis of strong ER-expression (Allred score of 7–8), the diagnostic performance of ^18^F-FES was improved. Few studies (Gupta et al. 2017; Peterson et al. 2011) yielded good correlation of ^18^F-FES and ER-expression in breast cancer, with higher ^18^F-FES uptake in higher ER-expressing tumor cells, resulting in an increased detection rate. In contrast, the diagnostic performance of ^18^F-FDG PET/CT was worsened due to less aggressive behavior in higher ER-expressing tumor cells (Mooij et al. 2023).

In the patient analysis, ^18^F-FDG and ^18^F-FES PET/CT revealed concordant results from TNM stage among 18 of 28 patients (64.29%). Most discordant results were from ^18^F-FDG PET/CT with 3 false-positive and 5 false-negative cases. Meanwhile, there was only 1 case with false negative ^18^F-FES PET/CT, showed superiority to ^18^F-FDG PET/CT for initial staging in ER-positive breast cancer. Interestingly, one case (Fig. 4) was both false-positive in ^18^F-FDG (contralateral axillary lymph nodes compatible with reactive nodes from recent vaccination that exhibited a decreased size in the follow-up imaging) and false-negative in ^18^F-FES (primary tumor and regional lymph nodes demonstrated by tissue diagnosis and anatomical findings). Hence, we propose that the combination of ^18^F-FDG and ^18^F-FES PET/CT could overcome the limitation of each radiotracer while improving the accuracy for initial staging.

Among semiquantitative parameters, ^18^F-FES had significantly higher SUVmax and T/N ratio than ^18^F-FDG PET/CT in lesions of ILC cell type, due to mostly strong ER expression (Xin and Eng 2016), low tumor density, low GLUT-1 expression, low proliferation rates, and infiltrative growth patterns (Fujii et al. 2016). ^18^F-FES PET/CT also had a significantly higher SUVmax and T/N ratio for Allred score 7–8 and Luminal A subtype. In contrast, ^18^F-FDG PET/CT had a significant higher SUVmax and T/N ratio in Allred score < 7 and Luminal B subtype. These results could be explained by the difference in the degree of ER-expression and tumor aggressiveness between each group (Peterson et al. 2008; Mooij et al. 2023).

In the multi-linear regression analysis, Allred score 7–8 significantly affected the degree of ^18^F-FES PET/CT with correlation coefficient of 5.90 (95%CI, − 0.0005, 11.81), *p* = 0.05, whereby higher ER expression was associated with higher ^18^F-FES uptake. Mucinous carcinoma also significantly affected the degree of ^18^F-FES uptake. This cell type usually has low aggressiveness and strong ER-expression (Hashmi et al. 2021), resulting in high ^18^F-FES uptake. However, this result originated from only one case and further study was recommended. Ki-67 is a marker of cell proliferation with good correlation to the degree of ^18^F-FDG PET/CT (Tchou et al. 2010) and is a critical criteria for categorize to the Luminal subtype. In our study, Ki-67 and Luminal B subtype were also noted to significantly affect the degree of ^18^F-FDG PET/CT, with correlation coefficient of 0.11 (95%CI, 0.01, 0.2), *p* = 0.025 and 2.76 (95%CI, 1.10,11.92), *p* = 0.018, respectively. In this study, BMI and menopause status yielded no significant effect to the degree of ^18^F-FES uptake, as in previous studies (Venema et al. 2016; Peterson et al. 2011). These results might be mainly due to the menopausal status (78.57%) and the normal BMI (67.86%) of the patients in this study.

## Conclusion

The overall diagnostic performance of ^18^F-FES is comparable to ^18^F-FDG PET/CT but has better diagnostic performance in Allred score 7–8. The combination of ^18^F-FDG and ^18^F-FES PET/CT can overcome the limitation of each radiotracer and improve diagnostic accuracy. Allred score of 7–8 is associated with a higher degree of ^18^F-FES PET/CT. Meanwhile, there is an association of Ki-67 and luminal B subtype with higher degrees of ^18^F-FDG PET/CT.

## Data Availability

The datasets generated during and/or analyzed during the current study are available from the corresponding author on reasonable request.

## Abbreviations

ER: Estrogen receptor
IHC: Immunohistochemistry
PET/CT: Positron emission tomography/Computed tomography
^18^F-FDG: ^18^F-Fluorodeoxyglucose
^18^F-FES: ^18^F-Fluoroestrodiol
BMI: Body mass index
VOI: Voxels of interest
SUVmax: Maximum standardized uptake value
T/N ratio: Tumor-to-normal ratio
MRI: Magnetic resonance imaging
SD: Standard deviation
IQR: Interquartile range
PPV: Positive predictive value
NPV: Negative predictive value
IDC: Invasive ductal carcinoma
ILC: Invasive lobular carcinoma
